# Non-Invasive Monitoring of Adrenocortical Activity in Three Sympatric Desert Gerbil Species

**DOI:** 10.3390/ani11010075

**Published:** 2021-01-04

**Authors:** Álvaro Navarro-Castilla, Mario Garrido, Hadas Hawlena, Isabel Barja

**Affiliations:** 1 Etho-Physiology Group, Unit of Zoology, Department of Biology, Faculty of Sciences, Autonomous University of Madrid, 28049 Madrid, Spain; isabel.barja@uam.es; 2 Jacob Blaustein Center for Scientific Cooperation, The Jacob Blaustein Institutes for Desert Research, Ben-Gurion University of the Negev, Midreshet Ben-Gurion 849900, Israel; gaiarrido@gmail.com; 3 Mitrani Department of Desert Ecology, Swiss Institute for Dryland Environmental and Energy Research, The Jacob Blaustein Institutes for Desert Research, Ben-Gurion University of the Negev, Midreshet Ben-Gurion 849900, Israel; hadashaw@bgu.ac.il; 4 Centro de Investigación en Biodiversidad y Cambio Global (CIBC-UAM), Autonomous University of Madrid, C/Darwin 2, 28049 Madrid, Spain

**Keywords:** adrenocorticotropic hormone, ACTH challenge, endocrine activity, enzyme immunoassay, fecal corticosterone metabolites, *Gerbil*, non-invasive monitoring

## Abstract

**Simple Summary:**

In this era, characterized by remarkable anthropogenic impacts on wildlife, it is crucial to monitor the health of wild animal populations while minimizing the interference to them. To this end, for a better understanding of the eco-physiology of wild animals, the adrenocortical activity can be non-invasively evaluated by measuring glucocorticoid metabolites excreted in feces. However, to ensure that the endocrine information is reliable, the experimental assays should be first validated and the causes for the major variability among individuals should be considered. Here we validated an enzyme immunoassay for measuring fecal corticosterone metabolites (FCM) in three wild gerbil species and emphasized the differences among them. These are endangered species, which play a key role in psammophilic communities, and provide a model system for various aspects in ecology. Thus, this work constitutes the first step toward using the FCMs of these species as indicators for individual and community stress.

**Abstract:**

The study of the endocrine status can be useful to understand wildlife responses to the changing environment. Here, we validated an enzyme immunoassay (EIA) to non-invasively monitor adrenocortical activity by measuring fecal corticosterone metabolites (FCM) in three sympatric gerbil species (*Gerbillus andersoni*, *G. gerbillus* and *G. pyramidum*) from the Northwestern Negev Desert’s sands (Israel). Animals included into treatment groups were injected with adrenocorticotropic hormone (ACTH) to stimulate adrenocortical activity, while control groups received a saline solution. Feces were collected at different intervals and FCM were quantified by an EIA. Basal FCM levels were similar in the three species. The ACTH effect was evidenced, but the time of FCM peak concentrations appearance differed between the species (6–24 h post-injection). Furthermore, FCM peak values were observed sooner in *G. andersoni* females than in males (6 h and 18 h post-injection, respectively). *G. andersoni* and *G. gerbillus* males in control groups also increased FCM levels (18 h and 48 h post-injection, respectively). Despite the small sample sizes, our results confirmed the EIA suitability for analyzing FCM in these species as a reliable indicator of the adrenocortical activity. This study also revealed that close species, and individuals within a species, can respond differently to the same stressor.

## 1. Introduction

In nature, wildlife is continuously and increasingly exposed to a wide variety of stimuli from environmental changes and anthropogenic perturbations. Consequently, animals have evolved behavioral responses and adaptations to face an ever-changing environment [1,2]. Observations of wildlife may be crucial for identifying factors associated with negative impacts (e.g., human activities or habitat alterations that may potentially disrupt wildlife behavior) and determine how affected or flexible individuals are to incipient changes. However, despite the advantages of direct monitoring in providing unique ecological insights on wildlife, its application is limited in certain species (e.g., rare and elusive species or those with large territories and/or low densities) and it may bias the results through human interference [3,4]. Furthermore, traditional techniques using direct monitoring are normally conditioned by logistical aspects and they are also both time and labor consuming [5]. Therefore, considering these budgetary and performance limitations, alternative methodologies to appropriately monitor animal population trends and their state of health are highly required.

In this regard, indirect indicators may play a decisive role in warning of the presence of external stimuli affecting wildlife populations. Human perturbations, environmental alterations and resource fluctuations are common sources of stress and may influence animal physiology to respond to such situations [6,7,8,9,10,11]. For example, upon facing stressful conditions, the typical response potentially involves the activation of the hypothalamic-pituitary-adrenal (HPA) axis leading to increased secretion of glucocorticoids (cortisol or corticosterone depending upon the species) into the bloodstream to maintain homeostasis [12,13]. Overall, while the temporary or short-term increase of glucocorticoids is considered to act as an adaptive response mediating the mobilization of energy stores to cope with stressors, chronic or long-term elevation is highly associated with detrimental effects on body condition, immunocompetence, reproductive output and survival [12,14,15,16]. In the past, the most widely used technique for measuring glucocorticoids was blood sampling [17]. However, besides being considered an invasive method, blood-based measures require animal capture and handling which may indeed increase the stress response [18]. In contrast, fecal sampling reliably samples occurrence of the study species and allows tracking animal populations. Additionally, measuring fecal glucocorticoid metabolites avoids disturbance to animals (e.g., handling/blood-sampling), so it has proved to be a suitable alternative to blood sampling [19]. Thus, to understand how stressors may affect animal populations and how individuals cope with them, measuring fecal glucocorticoid metabolites is a widely used non-invasive approach to quantify baseline levels and physiological responses of wildlife [20,21,22,23,24,25,26]. However, the analytical methods used must be appropriately validated for measuring fecal glucocorticoid metabolites and individual factors (e.g., sex) influencing their metabolism and excretion need to be controlled.

Gerbil communities are representative from the northwestern Negev desert’s sand in Israel and are mainly comprised of three species (*Gerbillus andersoni*, *G. gerbillus* and *G. pyramidum*). Wild gerbils have been used as a suitable model for investigating a vast array of ecological and behavioral aspects, including community dynamics, predation risk responses and host-parasite relationships [27,28,29,30,31,32]. Furthermore, according to the Red Book of Israel [33] the three gerbil species are considered as endangered species and they are also indicators for healthy sand communities, which are decreasing and are of conservation concern. These three species experience extreme fluctuating climatic conditions in the sandy desert environment, and evidence on their world distribution, habitat preference, and morphological characteristics suggest that *G. gerbillus* is the most adapted species to such fluctuating environmental conditions, followed by *G. pyramidum*, whereas *G. andersoni* is the least adapted species [34]. *G. gerbillus* is the rarest species and the only species that has major distribution shifts through time and space, occurring only sporadically in the Israeli parts of the sand dunes [27,28]. These three gerbils, as other wildlife species, can be also affected by other environmental and human factors acting as stressors [6,7,8,9,10,11]. However despite the great importance of understanding physiological aspects of wild animals, the few available works have been carried out only in *G. andersoni* [35,36,37,38] and *G. pyramidum* [16,36] and, as far as we know, currently there is no work addressing physiological stress responses in *G. gerbillus* nor is there any comparing physiological stress responses among these three sympatric species.

Cortisol is assumed to be the main glucocorticoid produced in some desert rodents [39,40,41,42], but according to Romero [8] corticosterone is the main glucocorticoid released in many rodent species and is the targeted glucocorticoid in gerbil species [16,35,36,43]. The main purpose of the present study was to optimize and validate the suitability of a commercial enzyme immunoassay for non-invasively monitoring adrenocortical activity, through measuring fecal corticosterone metabolites (FCM), in the above-mentioned three sympatric gerbil species of the Negev dunes. The optimization included the determination of the required time frame to detect FCM peak concentrations in fecal samples collected following a controlled stimulation of the HPA-axis via an adrenocorticotropic hormone (ACTH) injection. We also exploited the validated assay to get an initial exploration into species and gender differences in physiological stress responses.

## 2. Materials and Methods

### 2.1. Study Animals and Housing Conditions

We used 24 gerbils (*Gerbillus andersoni*: 6 males and 6 females; *G. gerbillus*: 6 males; *G. pyramidum*: 6 males). All animals belonged to the F1 offspring from parents who were previously captured from wild communities inhabiting the Northwestern Negev Desert´s sands in Israel. Only non-breeding adults were used to avoid bias in our results due to the potential influence of breeding condition. All animals were singly housed in standard plastic cages (34 × 24 × 13 cm) with a 1-cm layer of autoclaved sand as substrate. The animal facility was maintained with a 25 ± 1 °C temperature and with a constant diurnal rhythm of 12:12 h light-dark cycle, with artificial light from 07:00 to 19:00 for resembling natural light cycle. They were provided daily with millet seeds *ad libitum* and alfalfa as a water source according to Hawlena et al. [44]. 

### 2.2. Experimental Design

The experiment was conducted in October 2016. Before the experiment, the 24 gerbils were randomly assigned to either a treatment or a control group and moved to sampling cages 48 h before the experiment. On the day of the experiment, the treatment group received an intraperitoneal injection (a high dose of 60 μg/100 g of body weight of synthetic ACTH (Synacthen Depot, Novartis, Germany) [45]. Since an injection alone may also affect glucocorticoid concentrations [46,47], for controlling the potential effects of handling and injection procedures, control animals were treated the same, i.e., control groups in the three species were equally handled and injected with the same volume of isotonic saline solution. In order to avoid potential bias due to the time of the day influence in metabolism and excretion of glucocorticoids [47], all animals received the ACTH or the saline injection at the same time (between 16:50 h–17:25 h local time).

### 2.3. Faecal Sample Collection

Fecal sample collection was initiated before the experiment (i.e., pre-injection) to know baseline levels of fecal corticosterone metabolites (FCM). Concretely, feces were twice collected before the experiment, 48 h before and right before gerbils received the intraperitoneal injection (ACTH or saline solution depending on the groups assigned). During the experiment, fecal sample collection continued for 96 h post-injection at different time points (2, 4, 6, 9, 12, 18, 24, 36, 72, 96 h). This collection schedule allowed us to determine the time elapsed to detect any physiological stress reaction in feces. Fecal sample collection was randomized (changing the sampling order of individuals at each collection time point) to avoid bias and was done by the same person who ensured that only fresh and not urinated fecal samples were collected each time by removing any remaining feces from the sampling cage after each collection. Each collected fecal sample (consisting of several fecal pellets from the same individual) was frozen at −20 °C until FCM assays.

### 2.4. Measurement of Faecal Corticosterone Metabolites (FCM)

Fecal sample processing and FCM analysis in gerbil feces were performed at the Etho-Physiology Group’s laboratory (Autonomous University of Madrid) following the protocol from Navarro-Castilla et al. [45]. Briefly, fecal samples were unfrozen and dried in the heater until constant weight (at 90 °C 3 h). Later, 0.5 mL of phosphate buffer and 0.5 mL of 80% methanol were added to 0.5 g of dry feces in Eppendorf tubes. Tubes were vortexed 15 s and shaken on an orbital agitator overnight (16 h). Following this, samples were centrifuged for 15 min at 2500 rpm and fecal extracts were stored at −20 °C until analysis. Finally, FCM were quantified in the extracted samples via a DEMEDITEC Diagnostics corticosterone enzyme immunoassay (EIA), according to the kit specifications. This EIA used has been previously proven to be valid for measuring FCM in mammals, including rodent species [45,48]. 

The present EIA was biochemically validated by following previously described standard assay validation procedures (i.e., parallelism, accuracy and precision tests [49]). Parallelism assessment, through analyzing serial dilutions of fecal extracts (1:32, 1:16, 1:8, 1:4, 1:2, 1:1), yielded displacement slopes parallel to the respective slope of the calibration curve built with the standard corticosterone preparations provided by the manufacturer (*p* > 0.05). The recovery of exogenous corticosterone was performed by combining equal volumes of fecal extracts with known concentrations of exogenous corticosterone (standard preparations provided in the kit) and calculating the difference between the expected and observed FCM values (*n* = 6). Recovery was within 131.5 ± 24.0% accuracy. The precision of the EIA was assessed through intra and inter coefficients of variation (CV). The intra-assay CV (4.2%) was analyzed by running in the same plate fecal extracts (*n* = 6) in duplicate and the inter-assay CV (12.7%) was performed by running fecal extracts (*n* = 5) in four different plates. According to the manufacturer, the sensitivity of the corticosterone assay was >4.1 ng/mL and cross-reactivities of the antibodies with other substances are 12.5% for 11-deoxycorticosterone, 6.2% for progesterone, 2.3% for cortisol, 1.1 for pregnenolone and <1% with any other substance. In each assay, a blank and a control (standard provided by the manufacturer) were included. The assay was repeated if any standard exceeded 10% from the expected value. The biological validation of the EIA was performed through subjecting individuals from the three gerbil species to the ACTH challenge explained above in the experimental protocol. FCM values are presented in nanograms per gram of dry feces (ng/g).

### 2.5. Statistical Analyses

We firstly compared baseline FCM levels between collection time points before the experiment, i.e., 48 h before the experiment vs. right before the injection, fitting linear mixed models (LMM) to analyze repeated measurements. LMMs were performed separately for each species, setting FCM as the response variable and time point as a fixed factor. In the case of *G. andersoni*, LMMs were carried out both independently for each gender and combining data from both genders (sex included with time point as an interaction term). Finally, an LMM was performed for analyzing differences in basal FCM levels between the three gerbil species (fixed factor). 

To analyze physiological stress responses following the corresponding injections (ACTH/saline control), we ran independent LMMs for each species and, to simplify the analysis, data for ACTH and control groups were analyzed apart. In addition, despite we did not find differences in basal FCM levels due to gender, responses of *G. andersoni* males and females were also analyzed separately to avoid any potential bias in the results. In all LMMs performed, FCM was set as the response variable and time point (from 0 to 96 h) as a fixed factor. The highest FCM values detected were considered as the FCM peak concentrations and significant differences between FCM peak concentrations and basal values (right before the injection) were determined in a pairwise manner. To calculate FCM increase, individual baseline FCM values (right before the injection) were set as the 100% and FCM increase is expressed as a percentage of the respective basal value.

Animal ID was included in all LMMs as a random effect to account for pseudoreplication and control for interindividual variability. The response variable (FCM) was log-transformed to meet normality and homoscedasticity assumptions of model residuals (checked visually for each model by plotting the residuals as a function of fitted values [50]). Statistical analyses were done using the SPSS V22.0 software and results were considered significant at *p* < 0.05. Results are presented as mean ± standard error (SE) and coefficient estimates from models are given with their respective 95% confidence intervals (CIs).

## 3. Results

### 3.1. Baseline FCM Levels

Based on the results from the LMMs, we found no evidence to suggest differences in FCM levels between both collection time points before the experiment, i.e., 48 h before and right before injection (Table 1). In the case of G. andersoni, this result was found in both genders (males and females separately and combined). Furthermore, there were no gender differences in baseline FCM levels 48 h before the experiment nor right before the injection (LMM: *F*_2,12_ = 1.119, *p* = 0.359; *β* = −0.0991, 95% CI = −0.314, 0.115). Overall, we found no difference in baseline FCM concentrations between the three gerbil species at 48 h before starting the experiment (LMM: *F*_2,24_ = 0.940, *p* = 0.407) and right before the injection (LMM: *F*_2,24_ = 0.711, *p* = 0.502).

### 3.2. Experimental FCM Variation

Overall, we detected an increase in FCM values in the three gerbil species. Comparing mean FCM concentrations right before the injection, 1782 ng/g of dry feces (see values in Table 1), with mean FCM peak values of 7754 ng/g of dry feces detected during the experiment (range: 6610–9531 ng/g of dry feces), we found an average 358% increase in FCM values (range: 183–645%). However, the response to the stimulation differed markedly among groups since FCM peak concentrations occurred at different times (within 6–48 h following administration) depending on the species, treatment, and gender. 

**Figure 1:**
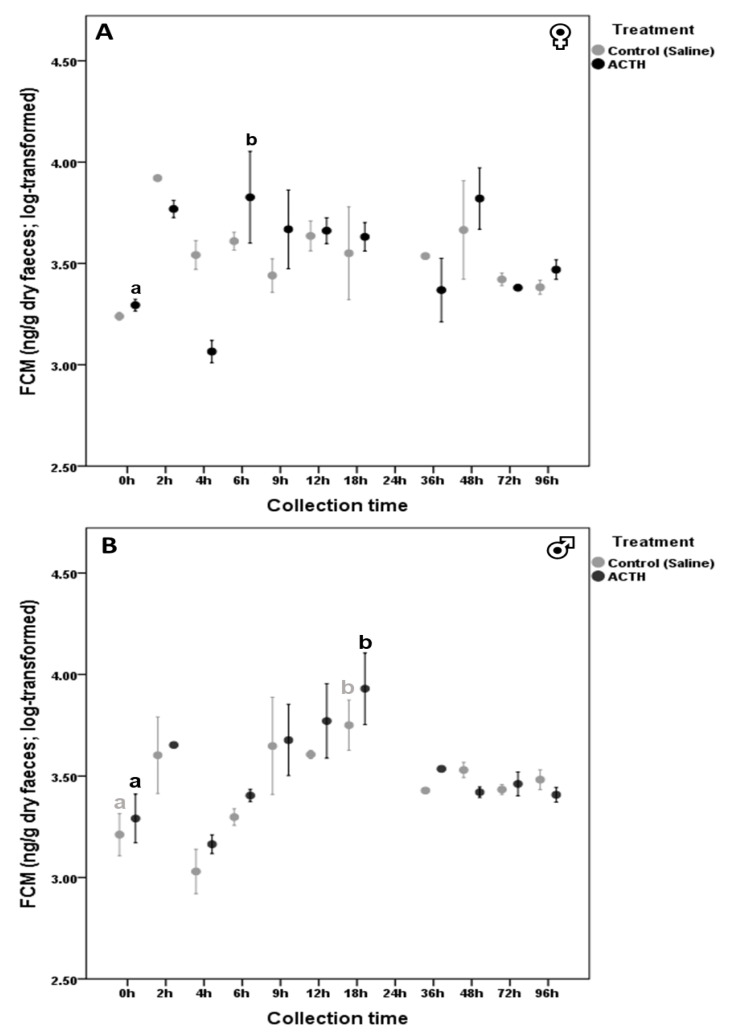
Fecal Corticosterone Metabolite variation (mean ± SE) in *G. andersoni* females (**A**) and males (**B**) after ACTH (adrenocorticotropic hormone)/saline solution injection (*n* = 3 per experimental group). Significant differences between basal (values at 0 h, i.e., right before the injection) and peak FCM (fecal corticosterone metabolites) values are indicated with different small letters.

In response to the ACTH challenge, both *G. andersoni* males (LMM: *F*_1,10_ = 7.309, *p* < 0.001) and females (LMM: *F*_1,10_ = 16.960, *p* = 0.008) showed increased FCM levels over time (Figure 1). However, significant FCM peak values were observed at 6 h in females (*β* = 0.532, SE = 0.131, 95% CI = 0.211, 0.912, *p* = 0.022; Figure 1A) and at 18 h in males (*β* = 0.639, SE = 0.174, 95% CI = 0.199, 1.079, *p* = 0.013; Figure 1B). Regarding control groups, females did not mount significant FCM peak concentrations (LMM: *F*_1,10_ = 1.528, *p* = 0.216; Figure 1A) but control males also responded to the saline solution injection (*F*_1,10_ = 6.003, *p* < 0.001; Figure 1B) showing a significant peak value 18 h following administration (*β* = 0.540, SE = 0.131, 95% CI = 0.216, 0.863, *p* = 0.007).

**Figure 2:**
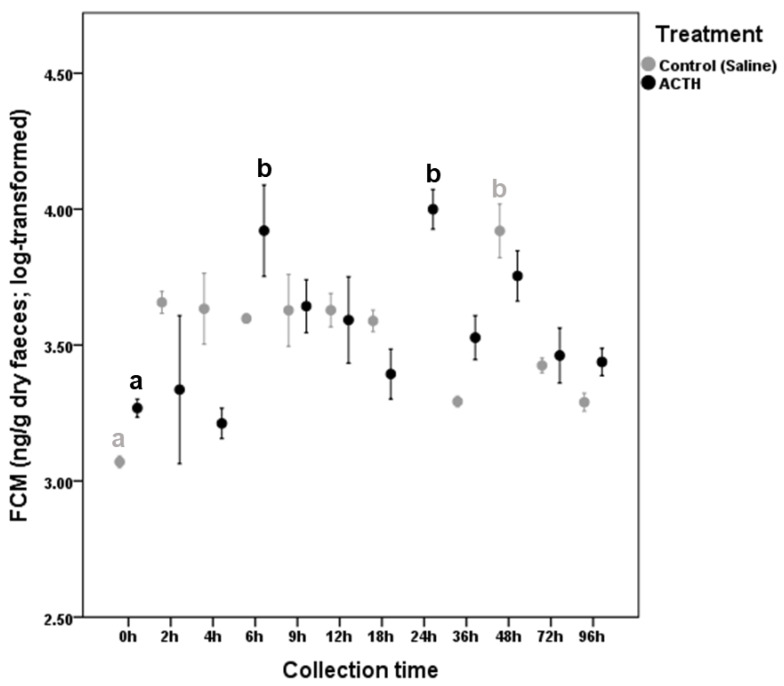
Fecal Corticosterone Metabolite variation (mean ± SE) in *G. gerbillus* (only males) after ACTH (adrenocorticotropic hormone) (*n* = 3) or saline solution (*n* = 3) injection. Significant differences between basal (right before the injection) and peak FCM (fecal corticosterone metabolites) values are indicated with different small letters.

*G. gerbillus* (males) experienced increased FCM levels after the ACTH (LMM: *F*_1,11_ = 4.248, *p* = 0.002), showing significant FCM peak concentrations at 6 h (*β* = 0.653, SE = 0.140, 95% CI = 0.224, 1.081, *p* = 0.016) and 24 h (*β* = 0.731, SE = 0.066, 95% CI = 0.553, 0.910, *p* < 0.001) following administration (Figure 2). In this species, the control group also mounted increased FCM levels (LMM: *F*_1,11_ = 4.248, *p* < 0.001) reaching a significant maximal value 48 h after the saline solution injection (*β* = 0.850, SE = 0.082, 95% CI = 0.600, 1.099, *p* = 0.001; Figure 2). 

**Figure 3:**
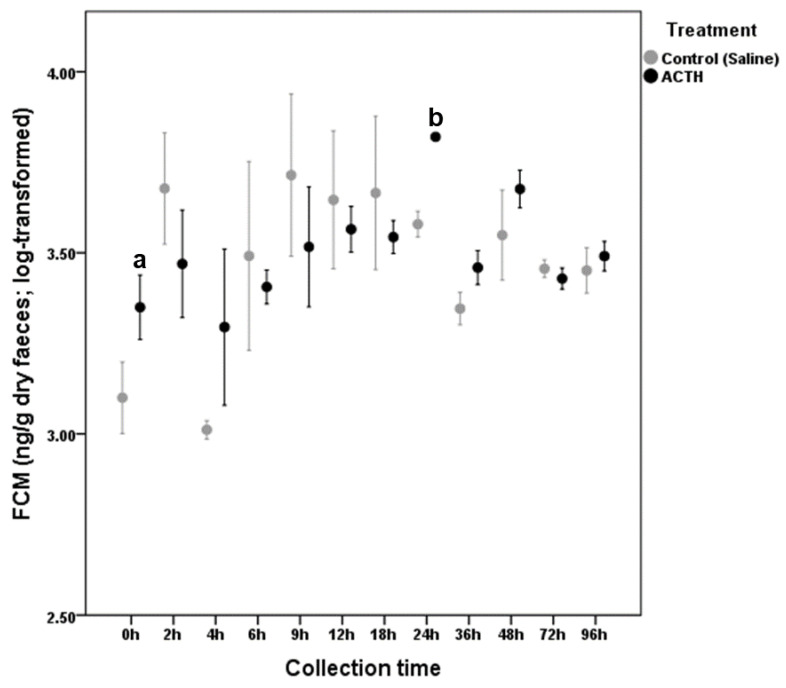
Fecal Corticosterone Metabolite variation (mean ± SE) in *G. pyramidum* (only males) after ACTH (adrenocorticotropic hormone) (*n* = 3) or saline solution (*n* = 3) injection. Significant differences between basal (right before the injection) and peak FCM (fecal corticosterone metabolites) values are indicated with different small letters.

*G. pyramidum* (only males) individuals showed virtually no difference between the ACTH and saline groups since both treatments evoked an increase in FCM levels (Figure 3). However, collection time did not result statistically significant for the saline control group (LMM: F_1,11_ = 1.881, *p* = 0.108). Conversely, the ACTH treatment lead to significant FCM peak concentrations (LMM: *F*_1,11_ = 2.784, *p* = 0.006) that were detected on feces 24 h after the ACTH injection (*β* = 0.471, SE = 0.073, 95% CI = 0.239, 0.702, *p* = 0.007; Figure 3).

## 4. Discussion

Considering that human pressure on the environment and wildlife continues leading to increasing concerns about biodiversity loss [51], there is an urgent need for appropriately monitoring wild animal populations, especially through non-invasive methods as early warning indicators of the physiological state of health. Our study confirmed that the ACTH experimental test rose fecal corticosterone metabolite (FCM) concentrations in the three gerbil species and proved the suitability of the corticosterone enzyme immunoassay (EIA) as a valid method for the non-invasive quantification of FCM concentrations in wild animals. Moreover, the indications for species- and gender-specific response dynamics and levels, despite the limited sample size and the relationships between the species, emphasize the importance of using individuals with variable characteristics for the efficient optimization and validation of assays. 

### 4.1. Baseline FCM Levels

Before the experimental procedure, variation in FCM concentrations between both initial measurements (i.e., 48 h and right before the injection) did not differ significantly in any of the three gerbil species. This stability in FCM values during the pre-experimental period suggests that there was not any effect of the sampling cage and accurately reflects baseline FCM levels of individuals. Furthermore, our findings indicated that the average baseline FCM concentration did not differ between the three gerbil species. This lack of difference in FCM basal values could probably be due to the closely related life-history patterns shared between the three species, because of experiencing similar environmental, ecological and evolutionary factors. Despite FCM, differences due to gender have been reported in other rodent species [21,39,52], here we found no evidence of significant intersexual differences in FCM levels in *G. andersoni*, which is consistent with results from other works carried out on this gerbils and other rodent species [36,39,53]. 

### 4.2. Experimental FCM Variation

The primary goal of this study was to validate the reliability of a commercially available EIA for non-invasively measuring FCM in three gerbil species. Consistent with this objective, the approximate four-fold increase in FCM values found in response to the ACTH challenge demonstrates the suitability of the present EIA for detecting FCM variations in these gerbil species. The average 358% increase in FCM found is comparable to the overall increase detected between basal and peak concentrations in gerbils (range: 220–374%; [16,36]). However, compared to these studies, FCM concentrations detected here are notably higher and probably due to differences in both the extraction protocol and the analytical means used. Variations in FCM values within the same species because of using different analytical procedures are commonly documented in the scientific literature, see for example basal FCM values found in the house mouse *Mus musculus domesticus* [46,54] or for the wood mouse *Apodemus sylvaticus* [7,55]. In this regard and considering the similar percentage of FCM increase obtained compared to the bibliography, the higher FCM values found might be partially supported by the EIA from Demeditec utilized in the present study, since it has been proved to quantify higher FCM concentrations than the Enzo´s EIA used in the studies by St. Juliana et al. [48]. 

By exploiting the validated assay, we had an initial exploration into species and gender differences in physiological stress. The different FCM variations detected along the experimental procedure provided valuable insight into some of the factors that may condition both specific and individual responses to environmental stressors. For example, although the three gerbil species increased FCM in response to the ACTH challenge, the response was detected sooner in *G. andersoni* (6–18 h), than in *G. gerbillus* (6–24 h) and *G. pyramidum* (24 h). Likewise, several research works have previously reported differences in physiological stress responses between co-occurring species [56], or even between sub-species [46]. Besides other interspecific factors possibly underlying differential stress-axis activation or conditioning the variability in the time course hormonal responses and their detection in feces, lag-time differences in FCM peak excretion between these species may be partially explained by differences in diet, metabolic rates and the intestinal passage time [57,58]. Compared to the existing data on gerbil species [16,36], overall, FCM peak values were detected later. For example, the time course of FCM excretion found for *G. andersoni* is congruent for females, but in males it exceeds the range previously found in this species (mean range: 5.5–9.25 h; [16,36]). Similarly, the time course of FCM peak excretion detected for *G. pyramidum* (24 h) is larger than the average 9.83 h found by St. Juliana et al. [16]. Animals from our experiment were all non-breeding individuals while St. Juliana et al. [36] do not indicate the reproductive status, differences in breeding condition of individuals may be, in part, behind the different results obtained. Moreover, St. Juliana et al. [36] neither indicated the time of the day when the experiment started, they just state “the night of the experiment”, and Touma et al. [47] shown that the time in FCM excretion can be significantly reduced during the night due to the influence of the circadian rhythm. 

Additionally, the saline control also increased FCM in males from *G. andersoni* and *G. gerbillus* but excretion time course and the return of FCM to nearly basal values differed between both species. In *G. andersoni* males, the time course for the excretion of FCM after the saline injection (18 h) was similar than the obtained with the ACTH test (18 h). In contrast, the post-saline FCM increase experienced by *G. gerbillus* males was detected at 48 h. These significant FCM elevations in response to saline injections may indicate that human handling and injection procedures may act as potential stressors for sensitive individuals. This result is in line with similar findings described in gerbils and other rodent species [36,46,54], where some individuals also exhibited FCM peaks in response to the saline injection used as the control.

Besides the indications for interspecific physiological responses, within the same species we found that, after the ACTH test, *G. andersoni* females displayed FCM peaks several hours prior to the males (females: 6 h; males: 18 h). Despite the above commented time elapse in FCM excretion, this intersexual variation was also reported by St. Juliana et al. [36]. In addition, as mentioned above, *G. andersoni* males also showed an increase in FCM values 18 h after the saline injection. Overall, gender-specific differences in FCM excretion are typically attributed to differences in the metabolism and/or excretion between both genders, mainly conditioned by differing effects of gonadal sex steroids, and their neuroactive metabolites, on the activity and responsivity of the HPA axis [59,60,61].

Altogether, these inter- and intra-specific variabilities in the course and level of the stress response, despite the small sample size of and relationship between the three gerbil species, emphasize the general importance of optimization and validation of non-invasive monitoring of adrenocortical activity for each new species. They also demonstrate the need to optimize and validate the response of individuals with different characteristics, despite the challenges that experiments with wild, sometime endangered, species, entail.

## 5. Conclusions

In this era, in which biodiversity is under a continuous risk, non-invasive monitoring of physiological stress responses of wild organisms has both theoretical and applied significance. Here we provided a validated method for non-invasively measuring FCM as a reliable indicator for assessing how free-ranging gerbils of these species cope and respond to alterations in their natural environment. To the best of our knowledge, our study represents the first attempt at analyzing endocrine activity in *G. gerbillus* and a valuable contribution to the previous studies conducted on the other two gerbil species. In particular, we collected some evidence suggesting that the course and level of physiological stress responses can differ among populations (e.g., differences between our results and the results of St. Juliana et al. [16,36]), closely related species, and even between sexes (current study). These complex responses to the same stressor could indicate that individuals may differently perceive stressors and accordingly undergo different hormonal adjustments to each stressful event. Notably, this context-dependence of endocrine responses highlights the key role of repeatability in science since, despite the laboratory standard conditions, the influence of different uncontrolled intrinsic variables may still yield ambiguous results. Therefore, these initial indications emphasize the importance of optimization and validation of non-invasive monitoring assays before any investigation of a new study system (e.g., new species or population) and encourage the inclusion of diverse individuals in each of the assays. Further studies including a large number of animals may help to control and curtail potential individual variation in stress-related responses.

## Figures and Tables

**Table 1:** Baseline fecal corticosterone metabolite (FCM) levels in the three gerbil species.

Species		Baseline FCM Levels (ng/g)		*F*	df	*p*	*β*	95% CI (Confidence Interval)
		48 h before	before Injection					
*Gerbillus andersoni*	males	1510.42	1920.00	1.046	1	0.336	−0.091	−0.278, 0.095
	females	1975.78	1857.33	0.184	1	0.683	0.008	−0.132, 0.148
	sexes combined	1743.10	1888.67	0.358	1	0.556	0.008	−0.143, 0.160
*G. gerbillus*	males	2842.33	1521.78	0.913	1	0.382	0.026	−0.197, 0.151
*G. pyramidum*	males	1711.28	1829.00	0.081	1	0.782	−0.009	−0.206, 0.188

## Data Availability

The data presented in this study are openly available in FigShare at https://doi.org/10.6084/m9.figshare.13514407.

## References

[1] Wong B., Candolin U. (2015). Behavioral responses to changing environments. Behav. Ecol..

[2] Gaynor K.M., Hojnowski C.E., Carter N.H., Brashares J.S. (2018). The influence of human disturbance on wildlife nocturnality. Science.

[3] Nowak K., le Roux A., Richards S.A., Scheijen C.P.J., Hill R.A. (2014). Human observers impact habituated samango monkeys’ perceived landscape of fear. Behav. Ecol..

[4] Blumstein D.T. (2016). Habituation and sensitization: New thoughts about old ideas. Anim. Behav..

[5] Verma A., van der Wal R., Fischer A. (2016). Imagining wildlife: New technologies and animal censuses, maps and museums. Geoforum.

[6] Wingfield J.C., Hunt K., Breuner C., Dunlap K., Fowler G.S., Freed L., Lepson J., Clemmons J.R., Buchholds R. (1997). Environmental stress, field endocrinology, and conservation biology. Behavioral Approaches to Conservation in the Wild.

[7] Navarro-Castilla Á., Mata C., Ruiz-Capillas P., Palme R., Malo J.E., Barja I. (2014). Are motorways potential stressors of roadside wood mice (*Apodemus sylvaticus*) populations?. PLoS ONE.

[8] Romero L.M. (2004). Physiological stress in ecology: Lessons from biomedical research. Trends Ecol. Evol..

[9] Cornils J.S., Hoelzl F., Huber N., Zink R., Gerritsmann H., Bieber C., Schwarzenberger F., Ruf T. (2018). The insensitive dormouse: Reproduction skipping is not caused by chronic stress in *Glis glis*. J. Exp. Biol..

[10] Łopucki R., Klich D., Ścibior A., Gołębiowska D., Perzanowski K. (2018). Living in habitats affected by wind turbines may result in an increase in corticosterone levels in ground dwelling animals. Ecol. Ind..

[11] Navarro-Castilla Á., Barja I. (2019). Stressful living in lower-quality habitats? Body mass, feeding behaviour and physiological stress levels in wild wood mouse populations. Integr. Zool..

[12] Sapolsky R.M., Romero L.M., Munck A.U. (2000). How do glucocorticoids influence stress responses? Integrating permissive, suppressive, stimulatory, and preparative actions. Endocr. Rev..

[13] McEwen B.S., Wingfield J.C. (2003). The concept of allostasis in biology and biomedicine. Horm. Behav..

[14] Romero L.M., Dickens M.J., Cyr N.E. (2009). The reactive scope model-a new model integrating homeostasis, allostasis, and stress. Horm. Behav..

[15] Sheriff M.J., Bosson C.O., Krebs C.J., Boonstra R. (2009). A non-invasive technique for analyzing fecal cortisol metabolites in snowshoe hares (*Lepus americanus*). J. Comp. Physiol. B.

[16] St. Juliana J.R., Khokhlova I.S., Wielebnowski N., Kotler B.P., Krasnov B.R. (2014). Ectoparasitism and stress hormones: Strategy of host exploitation, common host–parasite history and energetics matter. J. Anim. Ecol..

[17] Broom D.M., Johnson K.G. (1993). Stress and Animal Welfare.

[18] Place N.J., Kenagy G.J. (2000). Seasonal changes in plasma testosterone and glucocorticoids in free-living male yellowpine chipmunks and response to capture and handling. J. Comp. Physiol. B.

[19] Millspaugh J.J., Washburn B.E. (2004). Use of fecal glucocorticoid metabolite measures in conservation biology research: Considerations for application and interpretation. Gen. Comp. Endocrinol..

[20] Sánchez-González B., Planillo A., Navarro-Castilla Á., Barja I. (2018). The concentration of fear: Mice´s behavioural and physiological stress responses to different degrees of predation risk. Sci. Nat..

[21] Hernández M.C., Navarro-Castilla Á., Wilsterman K., Bentley G.E., Barja I. (2019). When food access is challenging: Evidence of wood mice ability to balance energy budget under predation risk and physiological stress reactions. Behav. Ecol. Sociobiol..

[22] Navarro-Castilla Á., Sánchez-González B., Barja I. (2019). Latrine behaviour and faecal corticosterone metabolites as indicators of habitat-related responses of wild rabbits to predation risk. Ecol. Ind..

[23] Ramahlo M., Chimimba C., Pirk C., Ganswindt A. (2019). Non-invasive monitoring of adrenocortical activity in free-ranging Namaqua rock mice *Micaelamys namaquensis* from South Africa in response to anthropogenic land use and season. Wildl. Biol..

[24] Piñeiro A., Barja I., Silván G., Illera J.C. (2012). Effects of tourist pressure and reproduction on physiological stress response in wildcats: Management implications for species conservation. Wildl. Res..

[25] Barja I., Silván G., Rosellini S., Piñeiro A., González-Gil A., Camacho L., Illera J.C. (2007). Stress physiological responses to tourist pressure in a wild population of European pine marten. J. Steroid Biochem..

[26] Barja I., Silván G., Illera J.C. (2008). Relationships between sex and stress hormone levels in feces and marking behavior in a wild population of Iberian wolves (*Canis lupus signatus*). J. Chem. Ecol..

[27] Abramsky Z., Rosenzweig M.L. (1984). Tilman’s predicted productivity–diversity relationship shown by desert rodents. Nature.

[28] China V., Kotler B.P., Shefer N., Brown J.S., Abramsky Z. (2008). Density-dependent habitat and patch use in gerbils: Consequences of safety in numbers?. Isr. J. Ecol. Evol..

[29] Bleicher S.S., Brown J.S., Embar K., Kotler B.P. (2016). Novel predator recognition by Allenby’s gerbil (*Gerbillus andersoni allenbyi*): Do gerbils learn to respond to a snake that can “see” in the dark?. Isr. J. Ecol. Evol..

[30] Messika I., Garrido M., Kedem H., China V., Gavish Y., Dong Q., Fuqua C., Clay K., Hawlena H. (2017). From endosymbionts to host communities: Factors determining the reproductive success of arthropod vectors. Oecologia.

[31] Kedem H., Cohen C., Messika I., Einav M., Pilosof S., Hawlena H. (2014). Multiple effects of host-species diversity on coexisting host-specific and host-opportunistic microbes. Ecology.

[32] Cohen C., Einav M., Hawlena H. (2015). Path analyses of cross-sectional and longitudinal data suggest that variability in natural communities of blood-associated parasites is derived from host characteristics and not interspecific interactions. Parasites Vectors.

[33] The Society for the Protection of Nature in Israel The Red Book of Israel. https://teva.org.il.

[34] Mendelssohn H., Yom-Tov Y. (1999). Mammalia of Israel.

[35] St. Juliana J.R., Kotler B.P., Wielebnowski N., Cox J.G. (2017). Stress as an adaptation I: Stress hormones are correlated with optimal foraging behaviour of gerbils under the risk of predation. Evol. Ecol. Res..

[36] St. Juliana J.R., Bryant J.L., Wielebnowski N., Kotler B.P. (2019). Physiological validation of a non-invasive method to evaluate adrenocortical activity and the time course for the excretion of stress hormones in the feces of three species of desert gerbils. Isr. J. Ecol. Evol..

[37] St. Juliana J.R., Kotler B.P., Pinshow B., Kronfeld-Schor N. (2019). Optimal foraging and physiological responses to the risk of predation: How fecal cortisol concentrations from trapped Allenby’s gerbil (*Gerbillus andersoni allenbyi*) relate to foraging under the risk of predation. Isr. J. Ecol. Evol..

[38] Eidelman A., Cohen C., Navarro-Castilla Á., Filler S., Gutierrez R., Bar-Shira E., Shahar N., Garrido M., Halle S., Romach Y. (2019). The dynamics between limited-term and lifelong coinfecting bacterial parasites in wild rodent hosts. J. Exp. Biol..

[39] Nováková M., Palme R., Kutalová H., Jansky L., Frynta D. (2008). The effects of sex, age and commensal way of life on levels of fecal glucocorticoid metabolites in spiny mice (*Acomys cahirinus*). Physiol. Behav..

[40] Frynta D., Nováková M., Kutalová H., Palme R., Sedláček F. (2009). Apparatus for collection of fecal samples from undisturbed spiny mice (*Acomys cahirinus*) living in a complex social group. J. Am. Assoc. Lab. Anim. Sci..

[41] Fraňková M., Palme R., Frynta D. (2012). Family affairs and experimental male replacement affect fecal glucocorticoid metabolites levels in the Egyptian spiny mouse *Acomys cahirinus*. Zool. Stud..

[42] Zatra Y., Aknoun-Sail N., Kheddache A., Benmouloud A., Charallah S., Moudilou E.N., Exbrayat J.M., Khammar F., Amirat Z. (2018). Seasonal changes in plasma testosterone and cortisol suggest an androgen mediated regulation of the pituitary adrenal axis in the Tarabul’s gerbil *Gerbillus tarabuli* (Thomas, 1902). Gen. Comp. Endocrinol..

[43] Kuznetsov V.A., Tchabovsky A.V., Kolosova I.E., Moshkin M.P. (2004). Effect of habitat type and population density on the stress level of midday gerbils (*Meriones meridianus* Pall.) in free-living populations. Biol. Bull..

[44] Hawlena H., Bashary D., Abramsky Z., Krasnov B.R. (2007). Benefits, costs and constraints of anti-parasitic grooming in adult and juvenile rodents. Ethology.

[45] Navarro-Castilla Á., Barja I., Díaz M. (2018). Foraging, feeding, and physiological stress responses of wild wood mice to increased illumination and common genet cues. Curr. Zool..

[46] Daniszová K., Mikula O., Macholán M., Pospíšilová I., Bímová B.V., Hiadlovská Z. (2017). Subspecies-specific response to ACTH challenge test in the house mouse (*Mus musculus*). Gen. Comp. Endocrinol..

[47] Touma C., Sachser N., Möstl E., Palme R. (2003). Effects of sex and time of day on metabolism and excretion of corticosterone in urine and feces of mice. Gen. Comp. Endocrinol..

[48] Abelson K.S., Kalliokoski O., Teilmann A.C., Hau J. (2016). Applicability of commercially available ELISA kits for the quantification of faecal immunoreactive corticosterone metabolites in mice. In Vivo.

[49] Taverniers I., De Loose M., Van Bockstaele E. (2004). Trends in quality in the analytical laboratory. II. Analytical method validation and quality assurance. Trends Anal. Chem..

[50] Singer J.D., Willett J.B., Willett J.B. (2003). Applied Longitudinal Data Analysis: Modeling Change and Event Occurrence.

[51] Mace G.M., Barrett M., Burgess N.D., Cornell S.E., Freeman R., Grooten M., Purvis A. (2018). Aiming higher to bend the curve of biodiversity loss. Nat. Sustain..

[52] Chelini M.O.M., Otta E., Yamakita C., Palme R. (2010). Sex differences in the excretion of fecal glucocorticoid metabolites in the Syrian hamster. J. Comp. Physiol. B.

[53] Scheibler E., Weinandy R., Gattermann R. (2004). Social categories in families of Mongolian gerbils. Physiol. Behav..

[54] Touma C., Palme R., Sachser N. (2004). Analyzing corticosterone metabolites in fecal samples of mice: A noninvasive technique to monitor stress hormones. Horm. Behav..

[55] Monarca R.I., da Luz Mathias M., Speakman J.R. (2015). Behavioural and physiological responses of wood mice (*Apodemus sylvaticus*) to experimental manipulations of predation and starvation risk. Physiol. Behav..

[56] Hammond T.T., Palme R., Lacey E.A. (2015). Contrasting stress responses of two co-occurring chipmunk species (*Tamias alpinus* and *T. speciosus*). Gen. Comp. Endocrinol..

[57] Palme R., Rettenbacher S., Touma C., El-Bahr S.M., Möstl E. (2005). Stress hormones in mammals and birds: Comparative aspects regarding metabolism, excretion, and noninvasive measurement in fecal samples. Ann. N. Y. Acad. Sci..

[58] Kalliokoski O., Jacobsen K.R., Teilmann A.C., Hau J., Abelson K.S. (2012). Quantitative effects of diet on fecal corticosterone metabolites in two strains of laboratory mice. In Vivo.

[59] Kudielka B.M., Kirschbaum C. (2005). Sex differences in HPA axis responses to stress: A review. Biol. Psychol..

[60] Reeder D.M., Kramer K.M. (2005). Stress in free-ranging mammals: Integrating physiology, ecology, and natural history. J. Mammal..

[61] Sze Y., Brunton P.J. (2020). Sex, stress and steroids. Eur. J. Neurosci..

